# KCNN4 is a Potential Biomarker for Predicting Cancer Prognosis and an Essential Molecule that Remodels Various Components in the Tumor Microenvironment: A Pan-Cancer Study

**DOI:** 10.3389/fmolb.2022.812815

**Published:** 2022-06-03

**Authors:** Shaohua Chen, Xiaotao Su, Zengnan Mo

**Affiliations:** ^1^ Department of Urology, The First Affiliated Hospital of Guangxi Medical University, Nanning, China; ^2^ Guangxi Key Laboratory for Genomic and Personalized Medicine, Center for Genomic and Personalized Medicine, Guangxi Collaborative Innovation Center for Genomic and Personalized Medicine, Guangxi Medical University, Nanning, China; ^3^ Department of Neurology, The Third Affiliated Hospital of Sun Yat-sen University, Guangzhou, China

**Keywords:** KCNN4, pan-cancer, tumor microenvironment, biomarker, tumor-infiltrating immune cells

## Abstract

**Objectives:** Potassium Calcium-Activated Channel Subfamily N Member 4 (KCNN4) is a member of the KCNN family. Studies have revealed that KCNN4 is implicated in various physiological processes as well as promotes the malignant phenotypes of cancer cells. However, little is known about its associations with survival outcomes across varying cancer types.

**Methods:** Herein, we systematically explored the prognostic value of KCNN4 in the pan-cancer dataset retrieved from multiple databases. Next, we performed correlation analysis of KCNN4 expression with tumor mutational burden (TMB) and microsatellite instability (MSI), and immune checkpoint genes (ICGs) to assess its potential as a predictor of immunotherapy efficacy. Afterwards, patients were divided into increased-risk group and decreased-risk group based on the contrasting survival outcomes in various cancer types. Furthermore, the underlying mechanisms of the distinctive effects were analyzed using ESTIMATE, CIBERSORT algorithms, and Gene Set Enrichment Analysis (GSEA) analysis.

**Results:** KCNN4 expression levels were aberrant in transcriptomic and proteomic levels between cancer and normal control tissues in pan-cancer datasets, further survival analysis elucidated that KCNN4 expression was correlated to multiple survival data, and clinical annotations. Besides, KCNN4 expression was correlated to TMB and MSI levels in 14 types and 12 types of pan-cancers, respectively. Meanwhile, different types of cancer have specific tumor-infiltrating immune cell (TICs) profiles.

**Conclusions:** Our results revealed that KCNN4 could be an essential biomarker for remodeling components in the tumor microenvironment (TME), and a robust indicator for predicting prognosis as well as immunotherapy response in pan-cancer patients.

## Introduction

Cancer is a serious disease which threatens human health and lowers life expectancy. According to statistics reported by the World Health Organization (WHO) in 2019, cancer was ranked as the first or second predominant cause of human death among people younger than 70 years in 112 out of 183 countries worldwide (Sung et al., 2021). In 2020, there were about 19,292,789 new cancer cases worldwide, with approximately 9,958,133 deaths. Notably, lung cancer is still the leading cause of cancer-related deaths, followed by colorectal cancer, liver cancer, stomach cancer, and female breast cancer (Sung et al., 2021). The discovery of immunotherapy introduced a new era into cancer treatment, and recent emergence of immune checkpoint inhibitors (ICIs) has revolutionized clinical cancer therapy (Billan et al., 2020). ICIs can block immune checkpoints pathway, thereby activating tumor-specific T cell immune responses and eliminating the cancer cells targeted by the immune system (Chen and Mellman, 2013). However, although clinical application of ICIs in recent years has presented encouraging efficacy in management of various tumors (Necchi et al., 2020), the treatment responses vary widely among different cancer types, along with individual differences in efficacy (Kelderman et al., 2014). Moreover, the adverse effects and the costs associated with the drugs pose significant challenges (Michot et al., 2016; Abdin et al., 2018). Therefore, this calls for identification of biomarkers to predict the prognosis and immunotherapy efficacy, with overarching goal of improving cancer treatment.

Potassium Calcium-Activated Channel Subfamily N Member 4 (KCNN4) is a member of the KCNN family, which encodes intermediate conductance potassium channel activated by intracytoplasmic calcium ions (Lin et al., 2020). Studies have revealed that KCNN4 is implicated in various physiological processes such as cell proliferation (Biasiotta et al., 2016), immune regulation (Chandy et al., 2004), and apoptosis (Simms et al., 2010). Notably, KCNN4 expression was upregulated in a variety of tumor tissues, including thyroid cancer (Wen et al., 2020), breast cancer (Zhang et al., 2016), and hepatocellular carcinoma (Du et al., 2019). In addition, aberrant KCNN4 expression was found to promote malignant phenotypes of cancer cells. Mo et al. (2021) reported that KCNN4 could induce progression and metastasis of pancreatic ductal adenocarcinoma (PDAC) through mesenchymal to epithelial transition factor (MET)-mediated AKT axis, whereas knockdown of KCNN4 resulted in apoptosis and cell cycle arrest in PDAC. These findings suggest its potential as a therapeutic target. Similarly, Lin et al. (2020) revealed that breast cancer progression and gemcitabine resistance could be a consequence of KCNN4 overexpression, suggesting that it could serve as a therapeutic strategy to overcome chemoresistance and improve the prognosis of breast cancer. However, it is worth noting that most studies on KCNN4 have been restricted to a single cancer type. Comparisons between diverse findings have been hampered by the sample sizes, methods for model construction, and the different experimental approaches. Therefore, there is an urgent need to extensively characterize the landscape of KCNN4 in pan-cancer datasets, which will provide novel insights for developing effective therapies for management of cancers.

**FIGURE 1:**
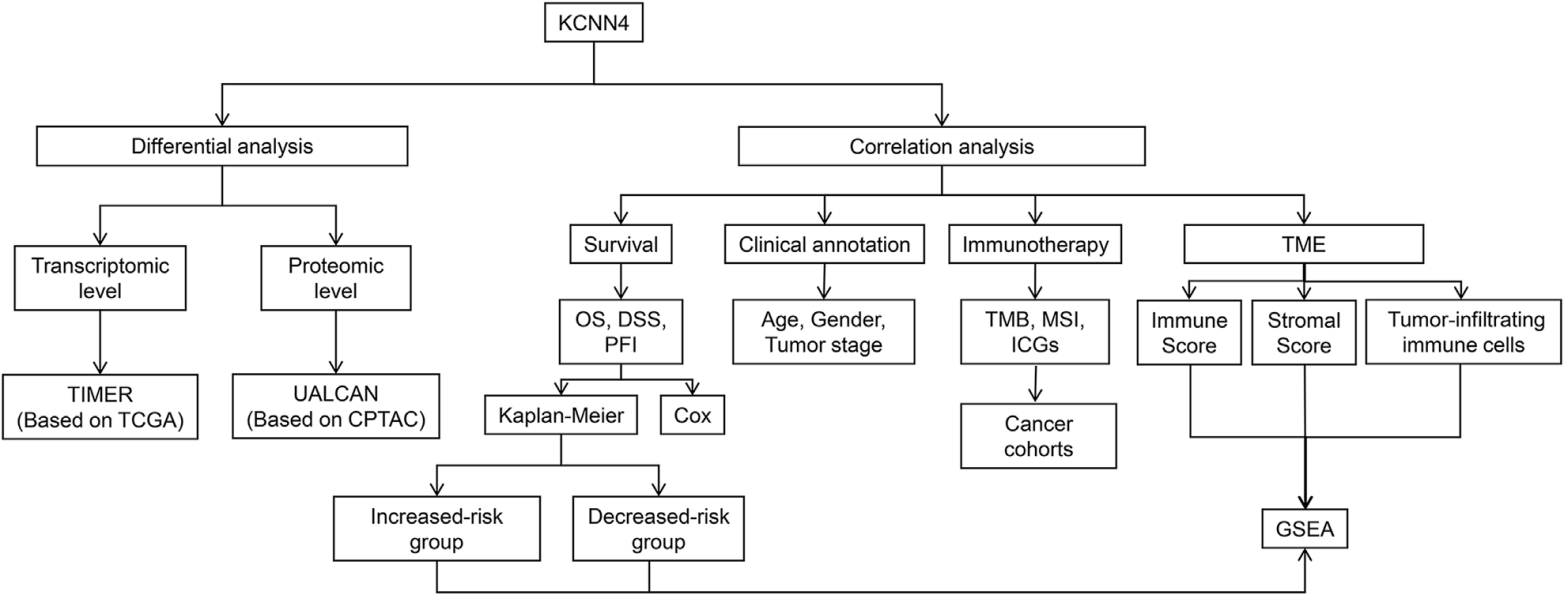
Analytical workflow of KCNN4.

Herein, we systematically dissected the prognostic value of KCNN4 in the pan-cancer dataset by combining multiple databases, including The Cancer Genome Atlas (TCGA), Tumor Immune Estimation Resource (TIMER), UALCAN, Clinical Proteomic Tumor Analysis Consortium (CPTAC), and cBioPortal databases. Correlation analysis of KCNN4 expression with tumor mutational burden (TMB), microsatellite instability (MSI), and immune checkpoint genes (ICGs) was then performed to explore its potential as a predictor of immunotherapy efficacy. Next, patients were divided into increased-risk, and decreased-risk groups based upon the contrasting survival outcomes in various cancer types. Furthermore, we elucidated the underlying mechanisms of the distinctive effects using ESTIMATE, CIBERSORT algorithms, and Gene Set Enrichment Analysis (GSEA). In summary, our findings revealed that KCNN4 is an essential identity for remodeling components in the tumor microenvironment (TME), and a robust biomarker for predicting prognosis and immunotherapy response in pan-cancer patients. Analytical workflow of the research is described in Figure 1.

## Materials and Methods

### Patient Information and Processing of Raw Data

RNA sequencing profiles, somatic mutations data, and associated clinical annotations for 33 types of human pan-cancer cohorts were downloaded from TCGA database using University of California Santa Cruz Xena (UCSC Xena; https://xena.ucsc.edu/) browser. The pan-cancer subtypes included the following: adrenocortical carcinoma (ACC), bladder urothelial carcinoma (BLCA), breast invasive carcinoma (BRCA), cervical squamous cell carcinoma and endocervical adenocarcinoma (CESC), cholangiocarcinoma (CHOL), colon adenocarcinoma (COAD), lymphoid neoplasm diffuse large B-cell lymphoma (DLBC), esophageal carcinoma (ESCA), glioblastoma multiforme (GBM), head and neck squamous cell carcinoma (HNSC), kidney chromophobe (KICH), kidney renal clear cell carcinoma (KIRC), kidney renal papillary cell carcinoma (KIRP), acute myeloid leukemia (LAML), brain lower grade glioma (LGG), lung squamous cell carcinoma (LUSC), liver hepatocellular carcinoma (LIHC), lung adenocarcinoma (LUAD), mesothelioma (MESO), ovarian serous cystadenocarcinoma (OV), prostate adenocarcinoma (PRAD), pancreatic adenocarcinoma (PAAD), pheochromocytoma and paraganglioma (PCPG), rectum adenocarcinoma (READ), stomach adenocarcinoma (STAD), sarcoma (SARC), skin cutaneous melanoma (SKCM), testicular germ cell tumors (TGCT), thyroid carcinoma (THCA), thymoma (THYM), uterine corpus endometrial carcinoma (UCEC), uveal melanoma (UVM), and uterine carcinosarcoma (UCS). The fragments per kilobase million (FPKM) values of RNA sequencing data downloaded from UCSC Xena were normalized as transcripts per kilobase million (TPMs) for the following analyses (Wagner et al., 2012; Abrams et al., 2019; Guo et al., 2022).

### Identification of Differential Expression Levels Between Pan-Cancer and Normal Samples

Differential expression analysis of KCNN4 between tumor and normal control tissues at the transcriptomic level was analyzed using TIMER database, which is an interactive web portal containing RNA sequencing profiles from TCGA database. Expression levels of KCNN4 were shown as log2 TPM value. In addition, comparison of protein expression patterns between tumor and normal control tissues were performed based on the UALCAN database, comprising proteomics data derived from the CPTAC database.

### Association of KCNN4 Expression With Survival Information of Pan-Cancer Patients

Kaplan-Meier curve analysis was performed to analyze correlation between KCNN4 expression and survival indicators, including overall survival (OS), disease-specific survival (DSS), and progression-free interval (PFI) of 33 types of pan-cancer patients. Subsequently, univariate Cox analysis was used to analyze the associations between KCNN4 expression and the above-mentioned survival indicators. Notably, “survival” (Therneau, 2015), “survminer” (Kassambara et al., 2018), and “forestplot” (Max Gordon and Lumley, 2020) R packages were used in the above analyses.

### Association of KCNN4 Expression With Clinical Annotations, Tumor Mutational Burden, and Microsatellite Instability in Pan-Cancer Patients

Somatic mutation data and clinical annotations of pan-cancer patients were downloaded from the TCGA database. For the clinical annotations such as age, gender, and tumor stage, we converted the clinical annotations into categorical variables, and compared the differences in KCNN4 expression levels between the two groups of patients with the Wilcoxon test *via* “limma” packages (Ritchie et al., 2015), and visualized by “ggpubr” (Kassambara, 2020) R package. Given the distribution pattern of the values and the variances between samples, Spearman’s correlation was used to analyze the correlation of KCNN4 expression with MSI and TMB was analyzed, and visualized as radar maps using “fmsb” (Nakazawa, 2018) R package.

### Association of KCNN4 Expression With Immune Components and Tumor-Infiltrating Immune Cell Profiles of Pan-Cancer Patients

To determine the proportion of immune components in the TME of pan-cancer samples, we used the ESTIMATE algorithm (Yoshihara et al., 2013) to calculate immune and stromal scores using “estimate” (Kosuke et al., 2021) and “limma” (Ritchie et al., 2015) R packages. Next, correlation analysis between KCNN4 expression and immune and stromal scores were performed. Moreover, relative tumor-infiltrating immune cells (TICs) levels were calculated *via* the CIBERSORT algorithm (Chen et al., 2018) and only tumor samples with *p* < 0.05 were retained for subsequent analysis. Similarly, correlation analysis between KCNN4 expression and relative TICs levels were carried out using Spearman’s correlation analysis, and visualized using “ggplot2” (Wickham, 2016), “ggpubr” (Kassambara, 2020), and “ggExtra” (Attali and Baker, 2019) R packages.

### Co-Expression Analysis of Immune-Related Genes and Gene Set Enrichment Analysis Analysis of KCNN4 in Pan-Cancer

Co-expression analysis between KCNN4 and immune-related genes was performed through “limma” (Ritchie et al., 2015) R package, further processing the data *via* “reshape2” (Wickham, 2007), and visualized by “RColorBrewer” (Erich, 2014) package. GSEA analysis was then carried out to decipher the mechanisms of KCNN4 in pan-cancers, and the top five enrichment terms of each tumor types were shown using the “clusterProfiler” (Yu et al., 2012) R package.

### Statistical Analysis

Transcriptomic expression patterns of KCNN4 between cancer and normal control tissues were compared through non-parametric Mann-Whitney test according to TIMER database. Comparisons of protein expression patterns and methylation levels of KCNN4 between tumor and normal control tissues were performed using Student’s t test based on the UALCAN database. Moreover, Kaplan-Meier curve analysis and univariate Cox regression analysis were performed to determine the association between KCNN4 expression and survival information of pan-cancer patients. *p* < 0.05 was considered statistically significant.

## Results

### Pan-Cancer Expression Patterns of KCNN4 in Transcriptomic and Proteomic Levels

**FIGURE 2:**
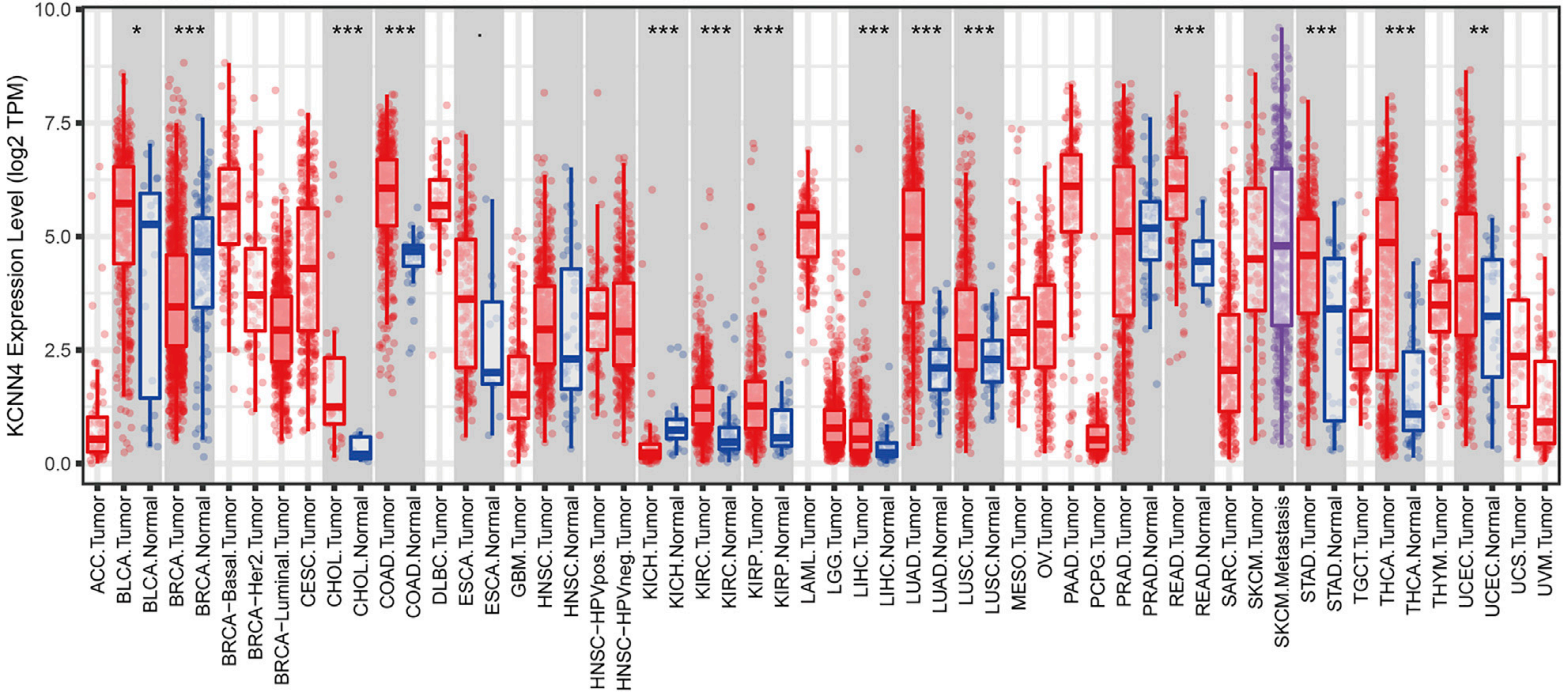
Expression patterns of KCNN4 in pan-cancer datasets based on TIMER database. ∗*p* < 0.05, ∗∗*p* < 0.01, ∗∗∗*p* < 0.001.

To evaluate the expression patterns of KCNN4, we performed differential analysis of KCNN4 expression between pan-cancer and normal control tissues at transcriptomic level based on TIMER database, and at proteomic level based on the UALCAN database. Results obtained in the TIMER database denoted that KCNN4 was significantly upregulated in various types of cancer tissues compared to normal control, including BLCA, CHOL, COAD, KIRC, KIRP, LIHC, LUAD, LUSC, READ, STAD, THCA, and UCEC. Conversely, the expression level of KCNN4 was diminished in BRCA and KICH tissues (Figure 2). In addition, KCNN4 protein expression was augmented in COAD and LUAD tissues compared to normal tissues, which was consistent with the transcriptomic profiles (Supplementary Figure S1). Notwithstanding, KCNN4 protein expression was upregulated in BRCA tissues, which was in contrast to the transcriptomic expression levels. Collectively, the results revealed the distinctively different KCNN4 expression patterns between cancer and normal samples in pan-cancer datasets.

### Genetic Alteration and Methylation Levels of KCNN4 in Pan-Cancer Types

**FIGURE 3:**
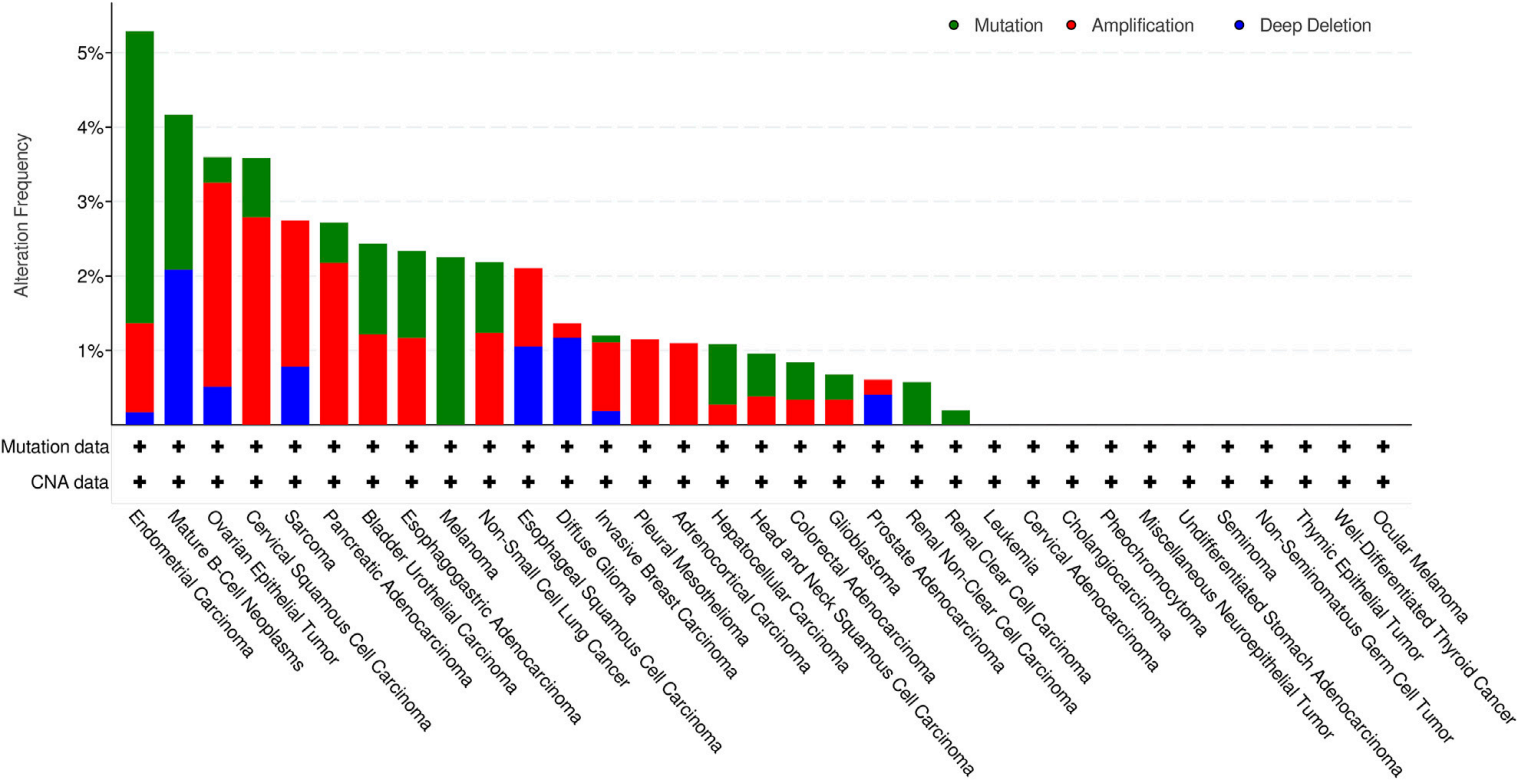
Alteration frequency of varying types of KCNN4 mutations.

Next, we investigated genetic alteration of KCNN4 based on cBioPortal database. Results indicated that KCNN4 was altered in 168 out of 10,508 patients, accounting for 1.6% of the total participants in the pan-cancer atlas studies. Amplification was the most predominant type of genetic alteration, followed by mutations and deep deletion. UCEC had the highest alteration frequency among the pan-cancer types (Figure 3). Subsequently, the methylation levels of KCNN4 between pan-cancer and normal control tissues were analyzed, indicating significant downregulation of methylation levels in tumor tissues compared to normal tissues in BLCA, BRCA, CESC, COAD, ESCA, HNSC, KIRC, KIRP, LIHC, LUAD, LUSC, PAAD, PCPG, READ, THCA, and UCEC, whereas PRAD presented higher levels of methylation in cancer tissues compared to normal control tissues (Supplementary Figure S2).

### Correlation Analysis Between KCNN4 Expression and Prognosis of Pan-Cancer Datasets

**FIGURE 4:**
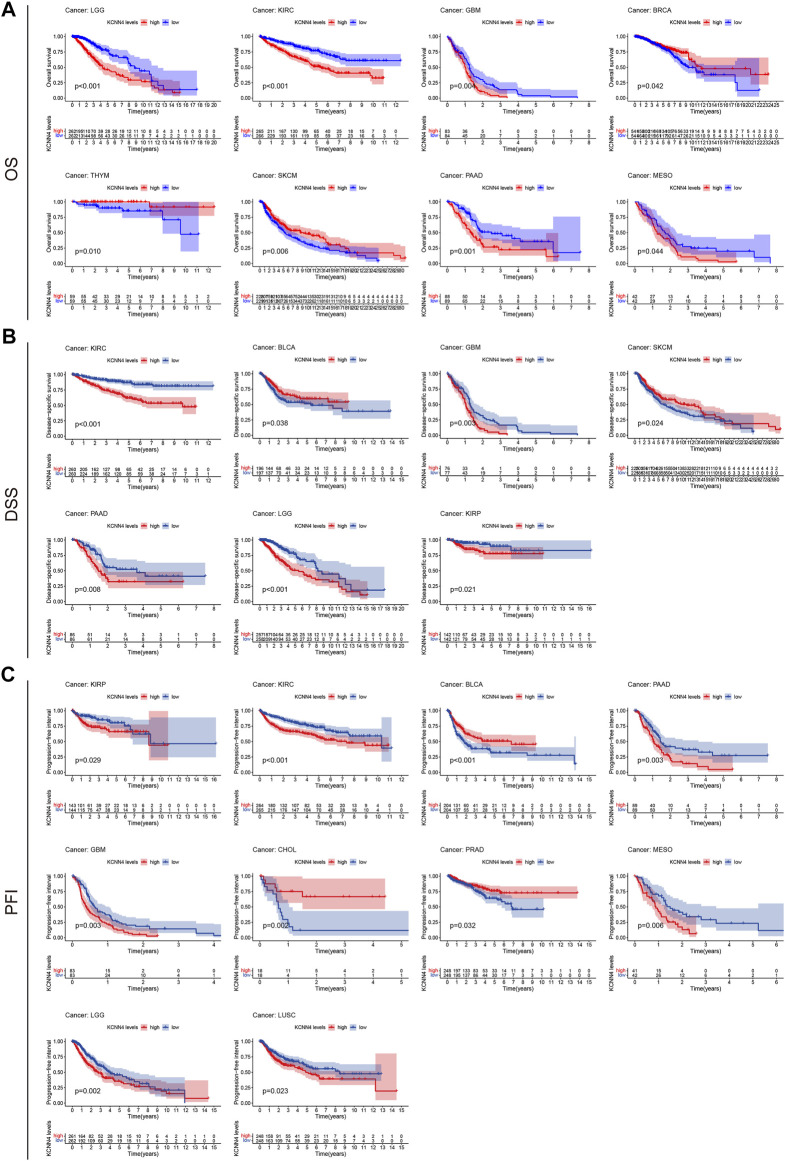
Correlation between KCNN4 expression level and survival indicators as determined through Kaplan–Meier curve analysis. **(A)** Correlation between KCNN4 expression level and OS as determined through Kaplan–Meier curve analysis. **(B)** Correlation between KCNN4 expression level and DSS as determined by Kaplan–Meier curve analysis. **(C)** Correlation between KCNN4 expression level and PFI as determined by Kaplan–Meier curve analysis.

We then analyzed the correlation between KCNN4 expression level and the survival indicators of pan-cancer patients, including OS, DSS, and PFI. The Kaplan–Meier curve analysis demonstrated that high expression of KCNN4 could exert unfavorable influences in the survival indicators (Figures 4A–C), including KIRP (DSS: *p* = 0.021; PFI: *p* = 0.029), LGG (OS: *p* < 0.001; DSS: *p* < 0.001; PFI: *p* = 0.002), MESO (OS: *p* = 0.044; PFI: *p* = 0.006), PAAD (OS: *p* = 0.001; DSS: *p* = 0.008; PFI: *p* = 0.003), GBM (OS: *p* = 0.004; DSS: *p* = 0.003; PFI: *p* = 0.003), LUSC (PFI: *p* = 0.023), and KIRC (OS: *p* < 0.001; DSS: *p* < 0.001; PFI: *p* < 0.001). Interestingly, high expression of KCNN4 played a protective role in six types of pan-cancers (Figures 4A–C), including SKCM (OS: *p* = 0.006; DSS: *p* = 0.024), THYM (OS: *p* = 0.01), BLCA (DSS: *p* = 0.038; PFI: *p* < 0.001); PRAD (PFI = 0.032), BRCA (OS: *p* = 0.042), and CHOL (PFI = 0.002).

**FIGURE 5:**
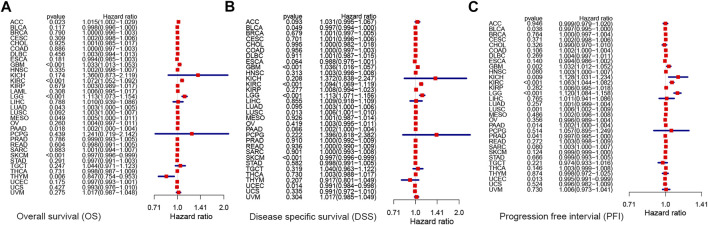
Correlation between KCNN4 expression level and survival indicators as determined through univariate Cox analysis. **(A)** Univariate Cox analysis of the correlation between KCNN4 expression level and OS. **(B)** Univariate Cox analysis of the correlation between KCNN4 expression level and DSS. **(C)** Univariate Cox analysis of the correlation between KCNN4 expression level and PFI.

Next, we performed univariate Cox analysis on the described survival indicators (Figures 5A–C). Results showed that KCNN4 could act as an adverse prognostic factor in GBM (OS: HR = 1.033, *p* < 0.001; DSS: HR = 1.036, *p* < 0.001; PFI: HR = 1.032, *p* = 0.002), KICH (PFI: HR = 1.128, *p* = 0.009), KIRC (OS: HR = 1.072, *p* < 0.001; DSS: HR = 1.094, *p* < 0.001; PFI: HR = 1.063, *p* < 0.001), LGG (OS: HR = 1.113, *p* < 0.001; DSS: HR = 1.113, *p* < 0.001; PFI: HR = 1.120, *p* < 0.001), LUAD (OS: HR = 1.003, *p* = 0.043), LUSC (DSS: HR = 1.006, *p* = 0.013; PFI: HR = 1.006, *p* = 0.001), MESO (OS: HR = 1.005, *p* = 0.049), ACC (OS: HR = 1.015, *p* = 0.018), PRAD (PFI: HR = 1.002, *p* = 0.014), and PAAD (OS: HR = 1.002, *p* = 0.018; PFI: HR = 1.002, *p* = 0.014). According to the univariate Cox analysis results, KCNN4 was also a positive prognostic factor in the following pan-cancer types (Figures 5A–C), containing BLCA (DSS: HR = 0.997, *p* = 0.049; PFI: HR = 0.997, *p* = 0.038), PRAD (PFI: HR = 0.997, *p* = 0.041), THYM (OS: HR = 0.847, *p* = 0.006), SKCM (OS: HR = 0.997, *p* < 0.001; DSS: HR = 0.997, *p* < 0.001), and UCEC (DSS: HR = 0.991, *p* = 0.014; PFI: HR = 0.995, *p* = 0.013).

Altogether, these results suggest that KCNN4 could be a potential biomarker for predicting the prognosis of cancer. In addition, KCNN4 may play a completely varying physiological role in various cancer types, thereby resulting in varying survival outcomes in patients.

### Correlation Analysis Between KCNN4 Expression and Clinical Annotations of Pan-Cancers

**FIGURE 6:**
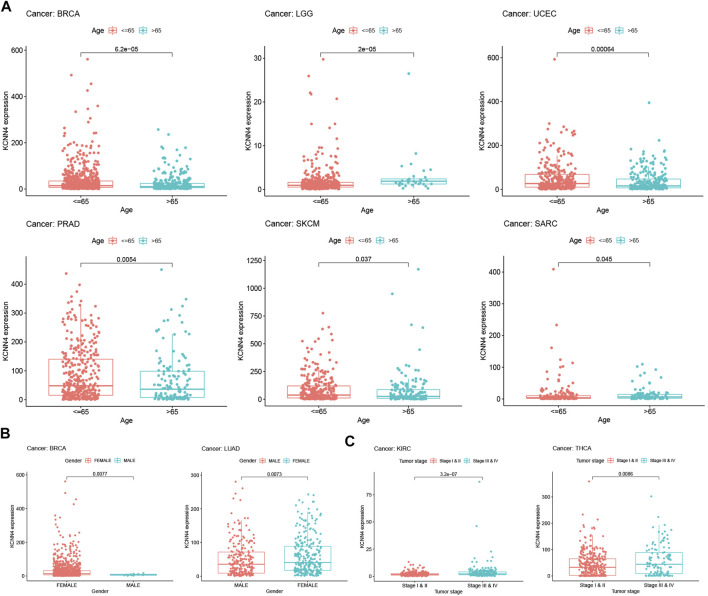
Correlation between KCNN4 expression level and clinical annotations. **(A)** Correlation between KCNN4 expression level and ages in SKCM, UCEC, PRAD, BRCA, LGG, and SARC. **(B)** Correlation between KCNN4 expression level and gender in BRCA, and LUAD. **(C)** Correlation between KCNN4 expression level and tumor stages in KIRC, and THCA.

The correlation between clinical annotations and KCNN4 expression was subsequently analyzed, with results confirming that KCNN4 expression was correlated with multiple clinical indicators. As shown in Figure 6A, patients younger than or equal to 65 years old had higher KCNN4 expression levels than patients older than 65 years old in SKCM, UCEC, PRAD, and BRCA cancers. In contrast, higher expression levels of KCNN4 were observed in patients older than 65 years in LGG and SARC cancers. Interestingly, KCNN4 expression also presented sex-specific differences in BRCA and LUAD (Figure 6B). Importantly, the expression levels of KCNN4 were significantly higher in stage III-IV patients than in stage I-II patients in THCA and KIRC cancers, suggesting that KCNN4 may reflect the clinical progression of these two tumors (Figure 6C).

### Correlation Analysis of KCNN4 Expression With Tumor Mutational Burden, Microsatellite Instability, and Immune Checkpoint Genes

**FIGURE 7:**
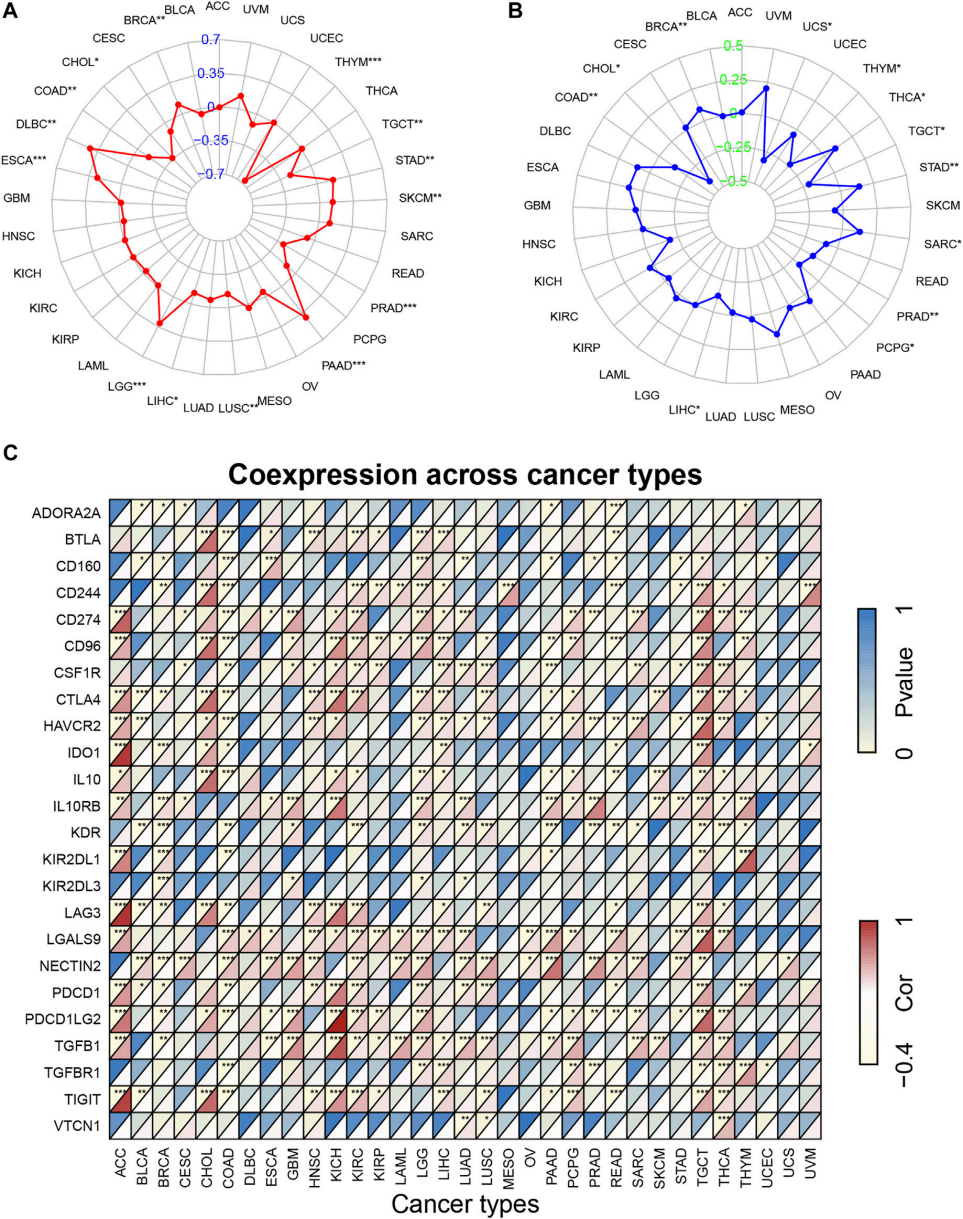
Correlation of KCNN4 expression with TMB, MSI, and ICGs. **(A)** Correlation between KCNN4 expression and TMB. **(B)** Correlation between KCNN4 expression and MSI. **(C)** Correlation of KCNN4 expression and ICGs.

We further explored the correlation of KCNN4 expression with TMB, MSI, and ICGs to determine the potential of KCNN4 as a biomarker for reflecting the efficacy of immunotherapy. TMB has been shown to be directly correlated with immunotherapy response in a variety of tumors (Yarchoan et al., 2017; Chan et al., 2019). We discovered that KCNN4 expression was correlated with TMB levels in 14 pan-cancer subtypes (Figure 7A). Specifically, KCNN4 expression levels were positively associated with TMB levels in BRCA, DLBC, ESCA, LGG, PAAD, SKCM, and STAD. In contrast, KCNN4 expression was negatively correlated with TMB levels in CHOL, COAD, LIHC, LUSC, PRAD, THYM, and TGCT.

Similarly, MSI is a recognized indicator that has a predictive value for the activity of immunotherapy (Dudley et al., 2016; Petrelli et al., 2020). Herein, we analyzed associations between the expression of KCNN4 and MSI levels in pan-cancer datasets (Figure 7B). Results revealed that KCNN4 expression was positively linked with MSI levels in BRCA, STAD, SARC, and THCA, and negatively linked with MSI levels in CHOL, COAD, LIHC, PCPG, PRAD, TGCT, THYM, and UCS.

Given that ICGs are a feasible indicator associated with efficacy of immunotherapy (Larkin et al., 2015; Chen and Mellman, 2017), we further performed correlation analysis between ICGs and KCNN4 expression in pan-cancer subtypes (Figure 7C). Notably, KCNN4 expression was significantly correlated with multiple ICGs, indicating positive correlation with most of the ICGs in LGG, TGCT, and THCA. From another point of view, KCNN4 expression was negatively correlated with a large portion of ICGs in BRCA and COAD. Besides, the results provided by Tumor Immune Dysfunction and Exclusion (TIDE) database (Jiang et al., 2018) indicated that KCNN4 has the moderate ability to predict clinical response of varying cancer cohorts treated with immunotherapy (Supplementary Figure S3), with 11 of the 21 immunotherapy-treated cohorts having an area under the receiver operating characteristic curve (AUC) greater than 0.5. Specifically, melanoma, and HNSC cohorts exhibited the strongest predictive likeliness of positive treatment responses for immunotherapy, with AUCs of 0.7229 and 0.65, respectively.

Overall, these results suggest that KCNN4 expression is correlated with TMB, MSI, and ICGs in multiple pan-cancer datasets, combined with evidence from TIDE, convincingly indicating that it could be a robust and reliable biomarker for predicting the responses of cancer cells to immunotherapy.

### Correlation Analysis KCNN4 Expression and Various Components in the Tumor Microenvironment of Pan-Cancer Types With Contrary Survival Outcomes

Studies have reported that immune and stromal components of the TME could affect cancer cell proliferation and response to therapy (Nakasone et al., 2012; Baghban et al., 2020). We aimed at unravelling the underlying mechanism of the contrary prognosis caused by KCNN4 *via* analyzing the components of TME in pan-cancer types. Therefore, the pan-cancers were divided into increased-risk group (four pan-cancer types) and decreased-risk group (two pan-cancer types) in according to the association between KCNN4 expression with survival outcome of pan-cancer patients obtained from Kaplan–Meier curve and univariate Cox analysis (Table 1). The ESTIMATE algorithm was then used to calculate the immune score and stromal score of the subjects in these six pan-cancer types, followed by analysis of their correlation with KCNN4.

**TABLE 1:** Univariate Cox analysis of the correlation between KCNN4 expression level and survival indicators.

Survival data	Increased-risk group (HR/*p*-Value)				Decreased-risk group (HR/*p*-Value)	
	LGG	PAAD	GBM	KIRC	SKCM	BLCA
OS	1.113/***	1.002/*	1.033/***	1.072/***	0.997/***	0.998/NS
DSS	1.113/***	1.002/NS	1.036/***	1.094/***	0.997/***	0.997/*
PFI	1.120/***	1.002/*	1.032/**	1.063/***	0.999/NS	0.997/*

NS, no significance. ∗*p* < 0.05, ∗∗*p* < 0.01, and ∗∗∗*p* < 0.001.

**FIGURE 8:**
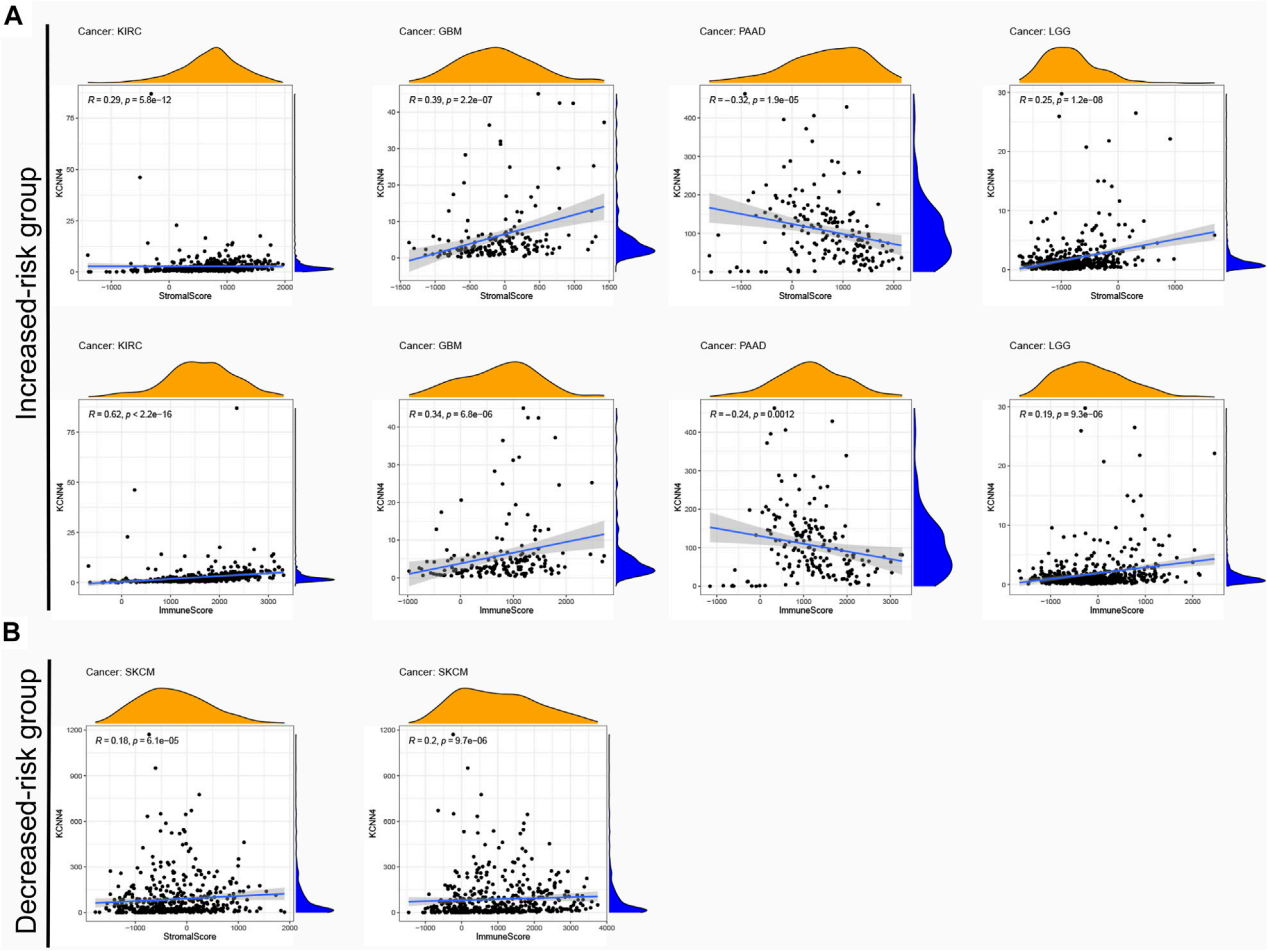
Correlation between KCNN4 expression and different components of TME. **(A)** Correlation between KCNN4 expression and different components of the increased-risk group. **(B)** Correlation between KCNN4 expression and various components of the decreased-risk group.

Notably, *p* < 0.05 was used as the cut-off value for correlation analysis. Results manifested that the immune scores of four pan-cancer types in the increased-risk group, including LGG, PAAD, GBM, and KIRC, were correlated with KCNN4 expression levels (Figure 8A). In addition, the immune scores of SKCM in the decreased-risk group was correlated with KCNN4 expression levels (Figure 8B). Unexpectedly, the immune score was positively correlated with KCNN4 expression in most of the tumor types in the two groups, and PAAD was the only pan-cancer type which was negatively correlated with KCNN4 expression. Similarly, the stromal score was positively correlated with KCNN4 expression in most pan-cancer types in both groups, with the exception of PAAD. Therefore, we hypothesized that KCNN4 could reprogram immune and stromal components of TME, thereby affecting the survival outcome of patients.

**FIGURE 9:**
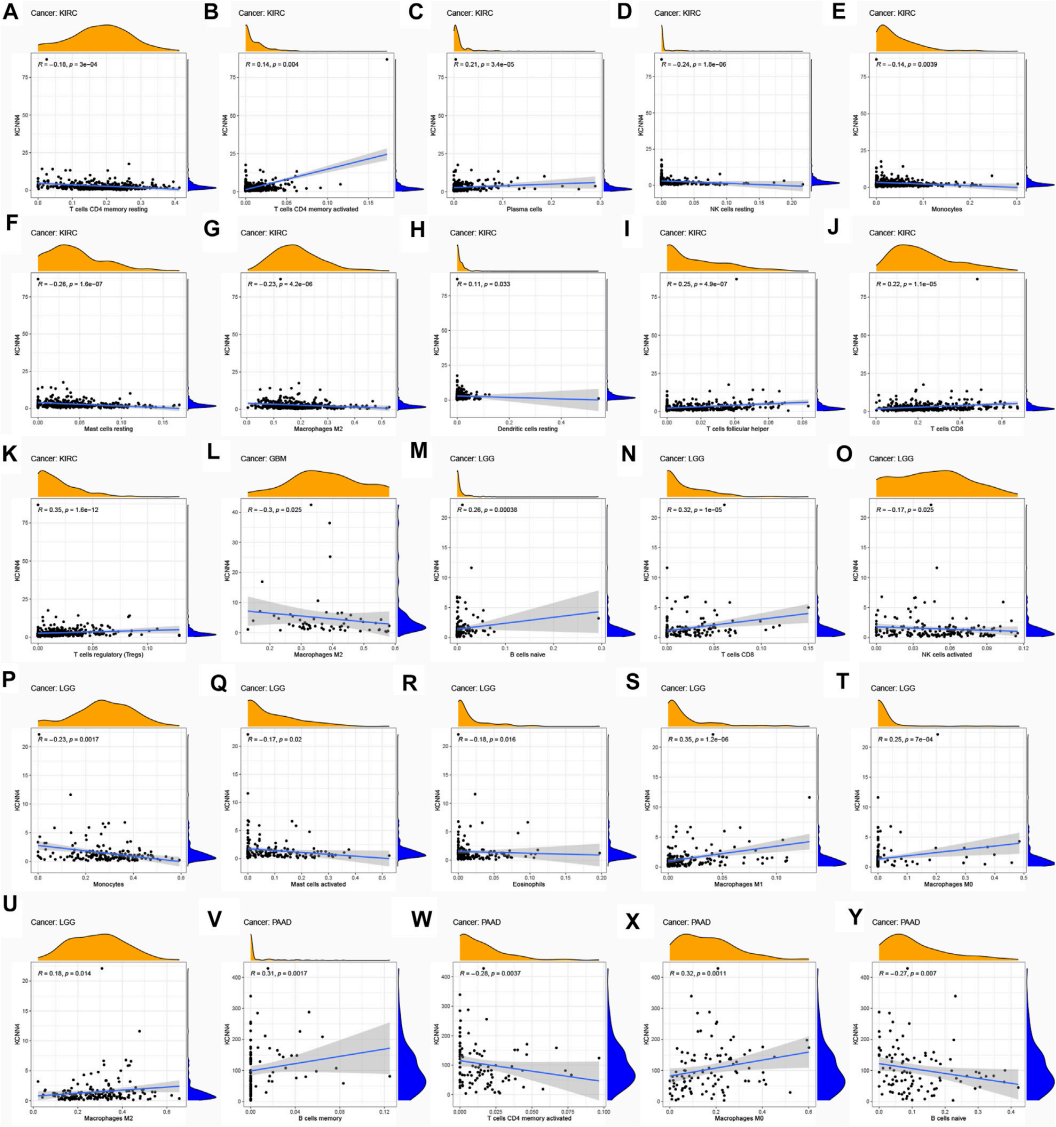
Association of TIC subtype with KCNN4 expression in increased-risk group. **(A–K)** Association of each TIC subtype with KCNN4 expression in KIRC. **(L)** Association of each TIC subtype with KCNN4 expression in GBM. **(M–U)** Association of each TIC subtype with KCNN4 expression in LGG. **(V–Y)** Association of each TIC subtype with KCNN4 expression in PAAD.

**FIGURE 10:**
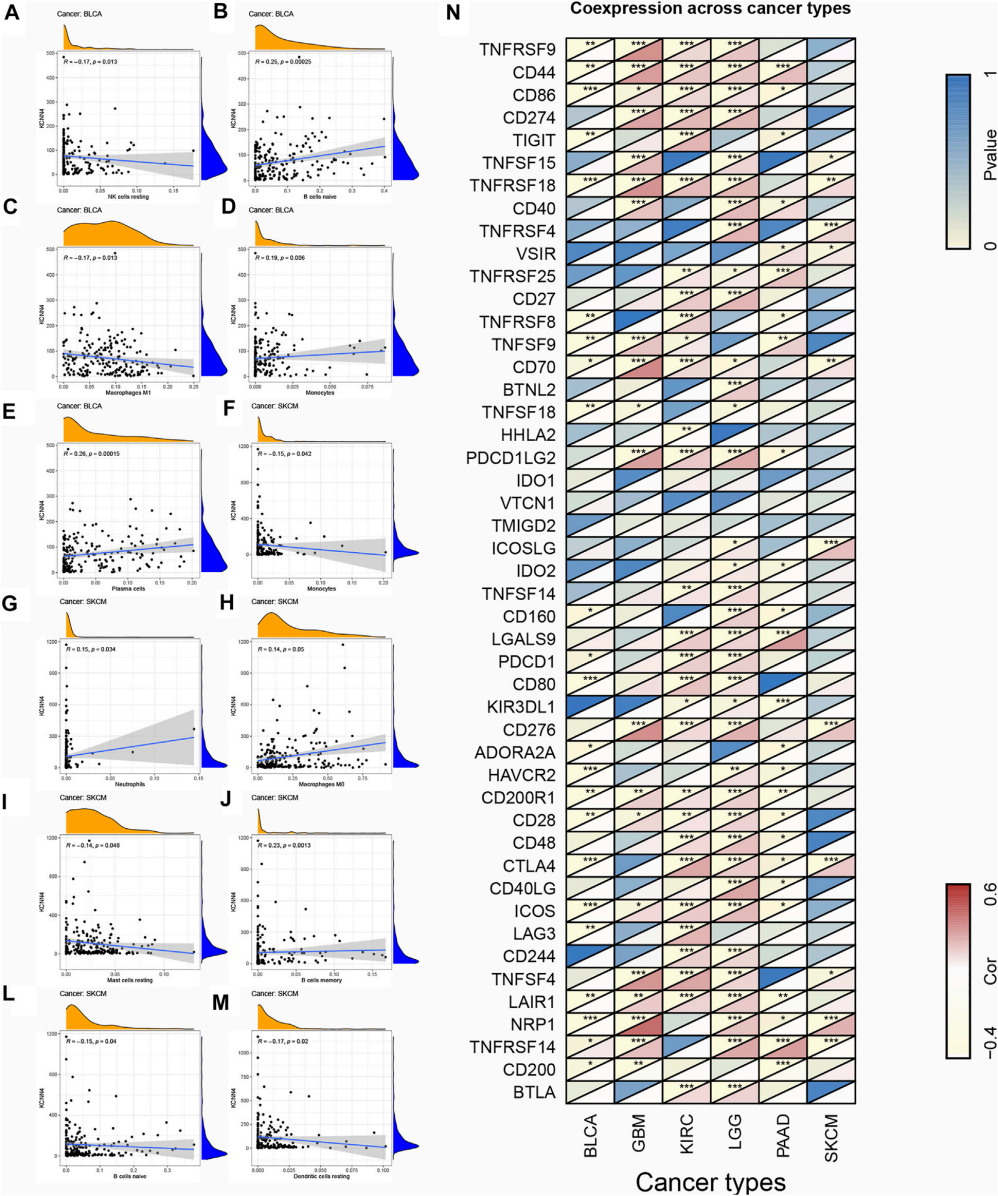
Association of TIC subtype with KCNN4 expression in decreased-risk group and the association of KCNN4 expression with immune-related genes. **(A–E)** Association of each TIC subtype with KCNN4 expression in BLCA. **(F–M)** Association of each TIC subtype with KCNN4 expression in SKCM. **(N)** Correlation between KCNN4 expression and immune-related genes.

To explore the association of each TIC subtypes with KCNN4 expression, the CIBERSORT algorithm was used to calculate the relative levels of TIC subtypes in patients in both groups, with *p* < 0.05 as the cut-off value. Interestingly, it was found that the immune components in the KIRC TME had the most profound effect of KCNN4, with a significant association between the levels of multiple TIC subtypes and KCNN4 expression (Figures 9A–K), and our results denoted that KCNN4 expression level was negatively linked with macrophage M2 in GBM (Figure 9L). In addition, KCNN4 expression levels were significantly positively correlated with macrophages (M0, M1, and M2), naïve B cells, and CD8 T cells in LGG, and negatively correlated with monocytes, activated NK cells, eosinophils, and activated mast cells. Furthermore, KCNN4 expression levels were positively linked with memory B cells, and macrophages M0, while negatively correlated with activated memory CD4 T cells and naïve B cells in PAAD. Significant associations also presented between immune components and KCNN4 expression in the decreased-risk group (Figure 10A). In summary, the results demonstrated that KCNN4 may exert its influence on tumor biological behavior by affecting various components in the TME, especially immune components. Thus, we performed a correlation analysis between KCNN4 and various immune-related genes (Figure 10B). Results revealed close interaction between KCNN4 and several immune-related genes, suggesting that KCNN4 may interfere with the balance between anti- and pro-tumor effects in the TME by influencing the expression of immune-related genes, which in turn mediates tumor progression and metastasis.

### Enrichment Analysis of Cancers in Different Groups

**FIGURE 11:**
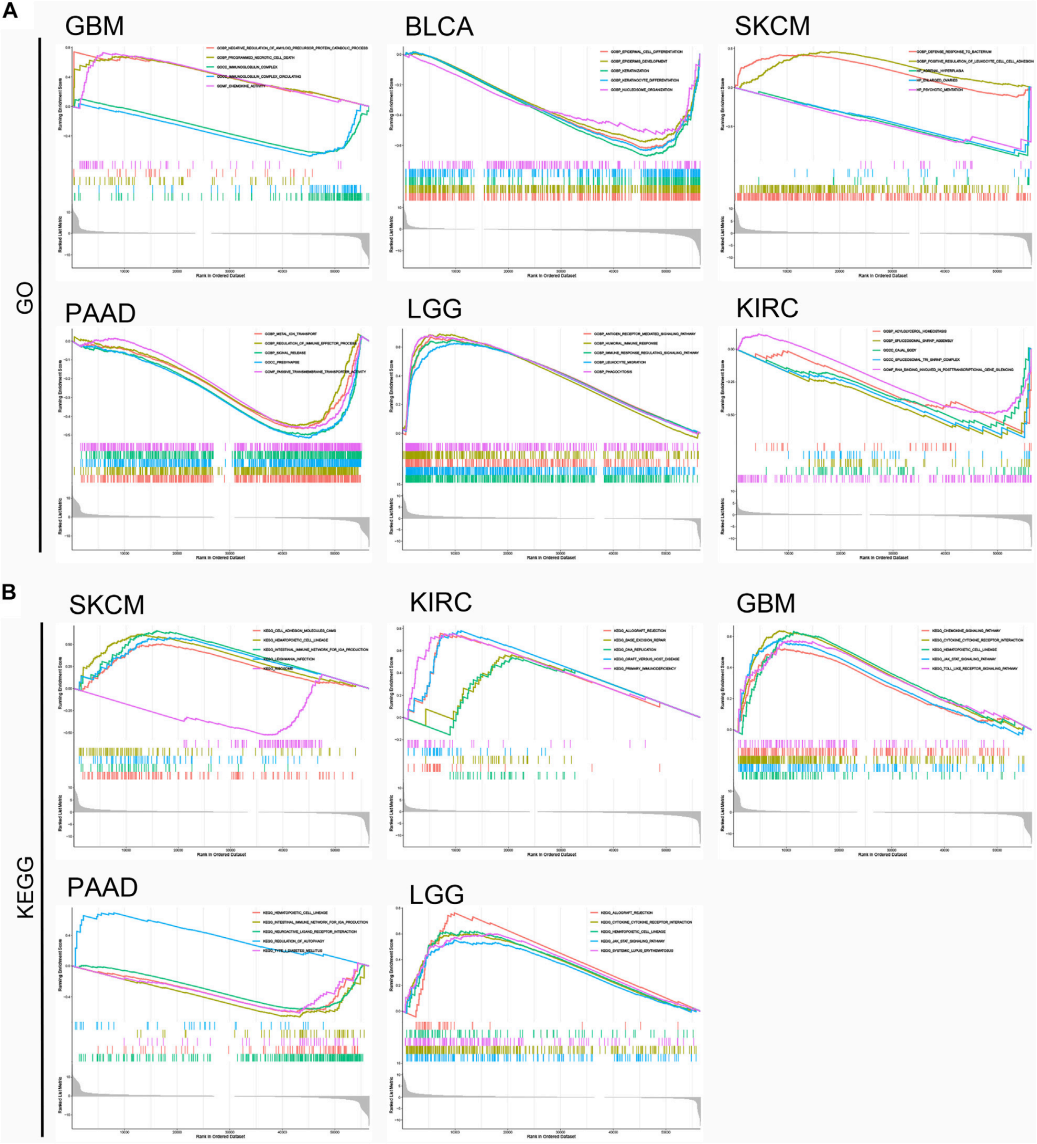
GSEA analysis of top functional terms associated with KCNN4 expression. **(A)** GO functional terms of KCNN4 in various cancer types in the increased-risk group and decreased-risk group. **(B)** KEGG pathway analysis of KCNN4 in various cancer types in the increased-risk group and decreased-risk group.

To further elucidate the potential molecular mechanism of KCNN4 in pan-cancers in the two groups, GSEA analysis was further performed on the six pan-cancer types (Figure 11). The results showed that KCNN4 expression was correlated with multiple immune-related ontology (GO) terms in the increased-risk group (Figure 11A), such as regulation of immune effector process in PAAD, chemokine activity in GBM, and leukocyte migration in LGG. Similarly, the expression of KCNN4 was involved in the positive regulation of leukocytes cell-cell adhesion in SKCM in the decreased-risk group, indicating that immune regulation may be one of the vital mechanisms through which KCNN4 plays a biological role.

Moreover, Kyoto Encyclopedia of Genes and Genomes (KEGG) analysis indicated that KCNN4 was mainly enriched in primary immunodeficiency of KIRC, intestine immune network for IgA production of PAAD, and cytokine-cytokine receptor interaction shared by GBM, and LGG (Figure 11B). Overall, these results demonstrated that KCNN4 was consistently enriched in cytokine, regulation of immune system, and inflammatory response related pathways, which confirms that KCNN4 acts as an essential identity for remodeling of the TME in various cancers.

## Discussion

The global burden of cancer incidence and cancer-related mortality has been progressively increasing (Bray et al., 2021). Such epidemiologic transition is an indication of both the accelerated aging of the population and the increased exposure to cancer-related risk factors (Gomes et al., 2020). The clinical use of new classes of anticancer drugs (e.g., targeted agents and ICIs) has led to improved survival rates in many cancer patients. Nevertheless, the heavy financial burden has negative implications on both the society and individuals (Howard et al., 2015; Savage et al., 2017). Per capita spending was reported to be four times higher for patients who received chemotherapy compared to those who did not (Nardi et al., 2016). Meanwhile, the exorbitant expenditure is not always bound up with clinical benefits in patients (Carrera et al., 2018). Therefore, implementing the concept of precision medicine, and identifying clinical biomarkers for predicting cancer prognosis and therapeutic response will provide valuable insights for cancer management and prevention.

Herein, we performed a comprehensive and systematic study of KCNN4 using pan-cancer datasets, confirming that its aberrant expression and significant downregulation of methylation levels in cancer tissues compared to non-cancerous tissues. Interestingly, the expression of KCNN4 in BRCA tissues was contradictory at the transcriptomic and proteomic levels, which may be attributed to the limited control sample sizes in proteomics or the involvement of post-transcriptional regulation. Besides, KCNN4 was significantly tied in with survival indicators and clinical annotations, including tumor stages in multiple cancer types, which suggests its potential role as a prognostic biomarker for cancers. Summarily, the role of KCNN4 in tumorigenesis has been supported by *in vitro*, and *in vivo* experiments, which is often recognized as an oncogene involving in the regulation of multiple cancer-related signaling pathways, thus mediating tumor progression, and development. Li et al. (2020) previously reported that KCNN4 could promote progression and invasion of hepatocellular carcinoma cells through mitogen-activated protein kinase (MAPK)/extracellular signal regulated kinase (ERK) pathway. Similarly, the association of KCNN4 with PI3K (Lin et al., 2020), endoplasmic reticulum stress (Yu et al., 2017), and TGF-β1 (Huang et al., 2013) signaling pathway have also been demonstrated. Note worthily, it has been well documented that KCNN4 is implicated in the evolution of resistance to anti-cancer therapies, as evidenced by Mohr et al. (2019) and Lin et al. (2020), indicating that KCNN4 could confer chemoresistance, and radioresistance in breast cancer, respectively.

The clinical development of immunotherapy has seen an unprecedented rise since the first ICIs were available. During the past several years, researchers have made great strides in identifying a robust biomarker for predicting efficacy of immunotherapy treatment (Gong et al., 2018). Studies have shown that MSI, defined as the collection of microsatellites mutations normally constituted by repeat length alterations and caused by deficient mismatch repair system (Vilar and Gruber, 2010), plays an essential role as a predictive biomarker for immunotherapy (Luchini et al., 2019). Pembrolizumab is a selective monoclonal antibody against PD-1, showing encouraging efficacy in multiple cancer types (Le et al., 2017). Results obtained from the clinical trials revealed that administration of pembrolizumab had moderate disease control rates and better survival outcomes in microsatellite instability-high (MSI-H) progressive metastatic colorectal cancer (Le et al., 2015). Similarly, high tumor mutational burden (TMB-H) was intimately linked to better objective response rates (ORR) and longer survival times after administration of immunotherapy (Yarchoan et al., 2017; Hellmann et al., 2018). Moreover, a recent study reported that TMB-H is a predictive marker for better OS in advanced gastric cancer patients who received toripalimab as a single agent (Wang et al., 2019). It was also correlated with clinical efficacy of pembrolizumab in the treatment of non–small cell lung cancer, manifesting as better ORR and PFI referred to as low tumor mutational burden (TMB-L) (Hellmann et al., 2018). Together with TMB and MSI, ICGs have been documented to be reliable biomarkers for predicting efficacy of immunotherapy. Expression of PD-L1 and PD-1 were the earliest recognized candidate biomarkers used to predict the efficacy of immunotherapy (Topalian et al., 2012; Taube et al., 2014; Doroshow et al., 2021), and were approved by the FDA for pre-treatment evaluation in patients with melanoma (Kaushik et al., 2022), bladder cancer (Roviello et al., 2021), gastric cancer (Huynh et al., 2021), and cervical cancer (Odiase et al., 2021). Moreover, transcriptomic and methylation levels of CTLA4 can be used for the same purpose in melanoma patients (Goltz et al., 2018; Mo et al., 2018), thereby providing critical references for selection of cancer patients likely to respond to ICIs. Similarly, other ICGs, including lymphocyte activation gene-3 (LAG-3), T cell immunoglobulin, and ITIM domain (TIGIT), have presented promising results in clinical trials (Andrews et al., 2017; Qin et al., 2019). Whereas, although great breakthroughs have been made in this field in the past years, each of the above-mentioned indicators has its limitations (Balar and Weber, 2017), which hampers selection of the most appropriate biomarker by oncologists. This study explored the possibility of using KCNN4 as a predictor of immunotherapy efficacy. It is noteworthy that KCNN4 expression was significantly correlated with TMB levels in 14 tumor types, with MSI levels in 12 tumor types, and with expression levels of multiple ICGs in several tumor types. Surprisingly, the expression levels of KCNN4 showed a consistently significant correlation with the above indicators in BRCA, COAD, LIHC, and TGCT. The results suggest that KCNN4 may have a higher accuracy in reflecting the response rate of immunotherapy for these cancer types. To sum up, these findings provide novel insights to break the current bottleneck of biomarker development in immunotherapy.

The TME is an intricate ecosystem made up of multiple immune cells and stromal cells. Notably, tumor progression and invasion result from the dynamic crosstalk between varying components in the TME (Wu and Dai, 2017), which is the predominant culprit for the development of drug resistance in anti-tumor therapies (Hinshaw and Shevde, 2019). Herein, results showed that cancer cells could reprogram the immune and stromal components of the TME, which indicates that they play a decisive role in tumor metastasis. It should be noted that the immune components release growth factors to promote the invasion of cancer cells (Goswami et al., 2005), whereas the stromal components secret extracellular matrix and cytokines (Räsänen and Vaheri, 2010), thereby contributing to therapeutic resistance (Paraiso and Smalley, 2013). Future studies should explore the dynamic changes of varying components in the TME, which will aid in identifying the immune status of the TME and elucidating the underlying mechanisms of tumorigenesis in certain tumor types. Herein, we found significant correlations between KCNN4 and immune scores as well as stromal scores in four tumor types in the increased-risk group, whereas KCNN4 expression was associated with immune and stromal scores of the SKCM in the decreased-risk group. Collectively, the above results suggest that KCNN4 may participate in remodeling the TME, which is consistent with results reported in a previous study (Brown et al., 2018; Chimote et al., 2018; Chen et al., 2021). Interestingly, we discovered that KCNN4 expression was correlated with various TIC subtypes and macrophages are the most frequently occurring cell types in different cancers. Xu et al. (2014) reported that the expression levels of KCNN4 could be upregulated in colorectal cancer cells co-cultured with tumor-associated macrophages (TAMs), which confirms that KCNN4 could be involved in inducing secretion of interleukins by TAMs and enhancing the malignant phenotypes of cancer cells**.** Overall, our findings demonstrated that KCNN4 may modulate the immune status of the TME *via* varying TIC subtypes and exerting influences in the survival outcomes of cancer patients, thereby unravelling the underlying mechanism of KCNN4 in the TME. Although there are certain pivotal conclusions obtained in our study, it has some limitations. First, it should be noted that the pan-cancer cohorts in this study only originated from TCGA database, and additional datasets are still imperative to further confirm our conclusions. Second, we concentrate on bioinformatics analysis, and *in vivo* and *in vitro* experiments should be performed to elucidate the mechanism by which KCNN4 affects TME. Third, as presented, the effect size produced by KCNN4 is relatively limited, suggesting that it may exert synergistic effects with other essential genes in TME to influence tumorigenesis, but the underlying mechanisms have not been fully elucidated.

## Conclusion

In summary, the results obtained in this study suggest that KCNN4 is a biomarker that can predict the prognosis of several cancer types. In addition, KCNN4 is associated with TMB, MSI, and varying components in TME, which confirms its potential for predicting the efficacy of immunotherapy treatment and immune status in TME. The findings of this study will provide insights for developing and expanding novel targets for further cancer management and prevention.

## Data Availability

The datasets presented in this study can be found in online repositories. The names of the repository/repositories and accession number(s) can be found in the article/Supplementary Material.

## Supplementary Material

The Supplementary Material for this article can be found online at: https://www.frontiersin.org/articles/10.3389/fmolb.2022.812815/full#supplementary-material

Supplementary Figure S1 Analysis of KCNN4 expression patterns at the proteomic level between pan-cancer and non-cancerous samples using CPTAC datasets from UALCAN database. **p* < 0.05, ***p* < 0.01, ****p* < 0.001.

Supplementary Figure S2 DNA methylation level of KCNN4 in pan-cancer datasets obtained from the UALCAN database. **(A–Q)** Analysis of DNA methylation level of KCNN4 between pan-cancer and non-cancerous samples. **p* < 0.05, ***p* < 0.01, ****p* < 0.001.

Supplementary Figure S3 Biomarker analysis of KCNN4 compared to other canonical biomarker signatures in immunotherapy-treated cancer cohorts based on the TIDE database.
