# Supplementary figures and images for: KCNN4 is a Potential Biomarker for Predicting Cancer Prognosis and an Essential Molecule that Remodels Various Components in the Tumor Microenvironment: A Pan-Cancer Study
Source: Front Mol Biosci. 2022 Jun 3;9:812815. doi: 10.3389/fmolb.2022.812815 (PMC9205469; doi:10.3389/fmolb.2022.812815)

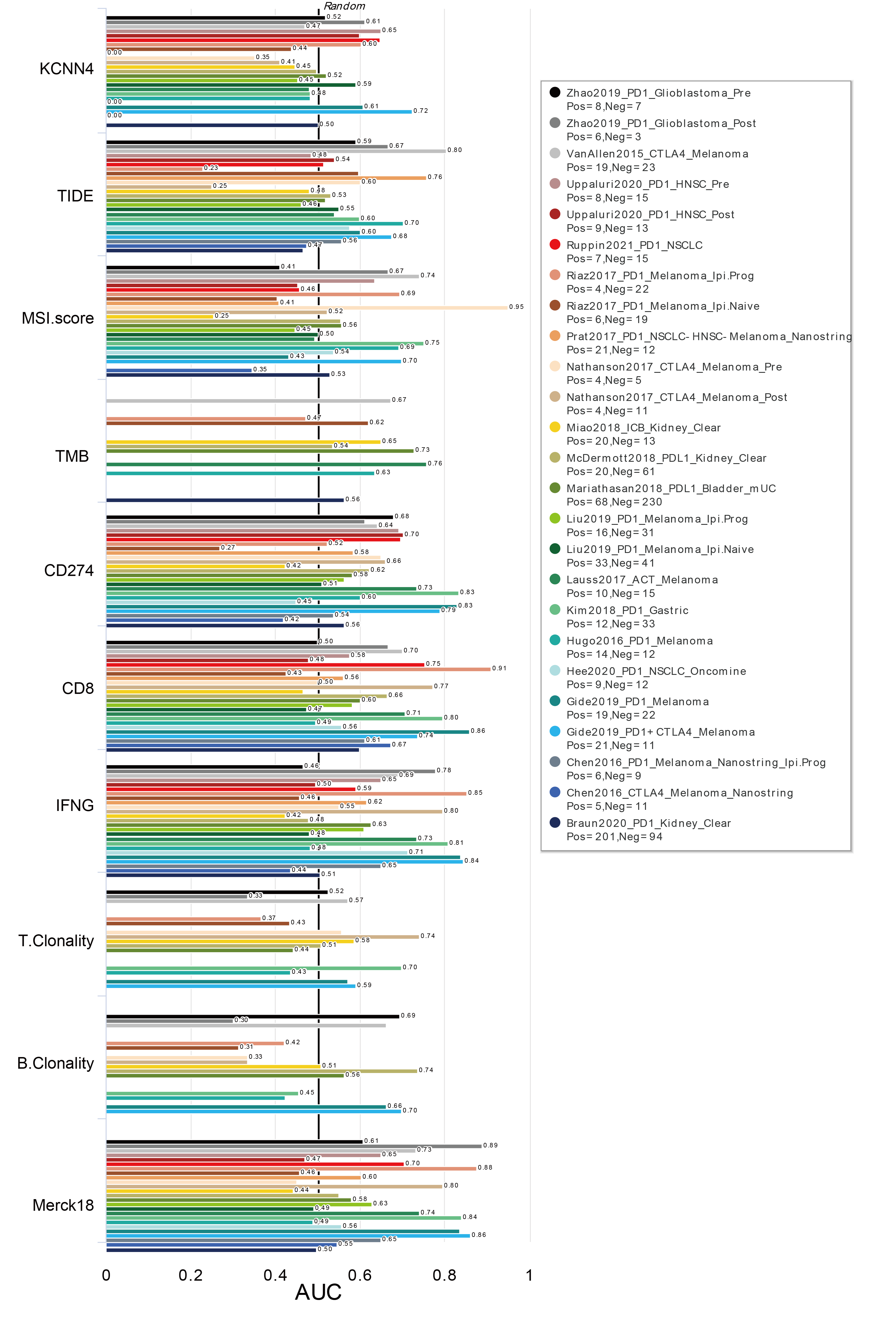

Supplement: Supplementary file 1 [file Image3.tif]

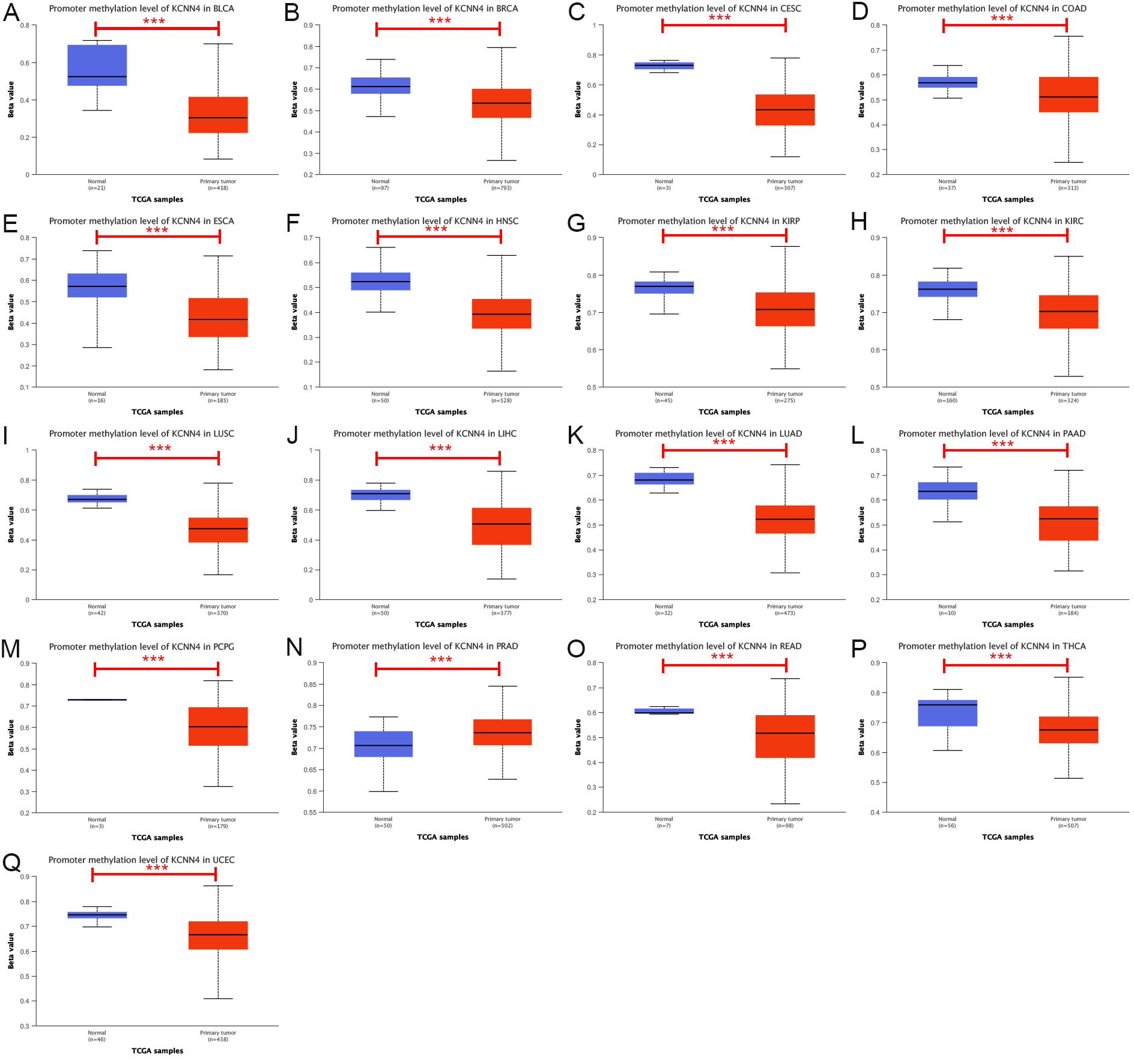

Supplement: Supplementary file 2 [file Image2.tif]

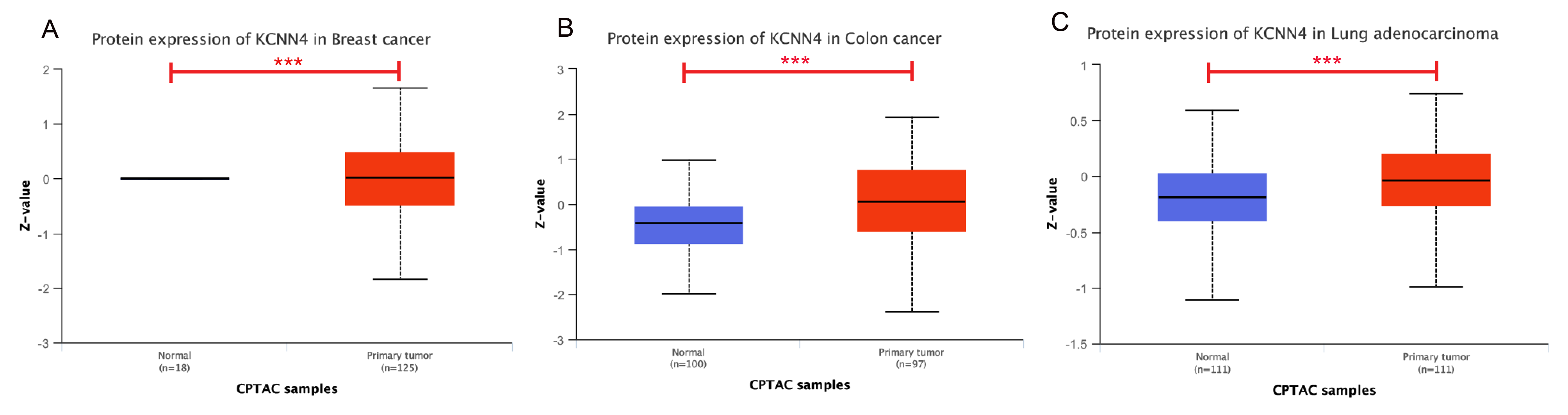

Supplement: Supplementary file 3 [file Image1.tif]
